# Clinical predictors of response to atezolizumab/bevacizumab in Child-Pugh B patients with hepatocellular carcinoma

**DOI:** 10.1016/j.jhepr.2026.101915

**Published:** 2026-06-11

**Authors:** Anne-Victoire Odent, Claudia Campani, Mohamed Bouattour, Yann Touchefeu, Victoria Delhoume, Olivier Rosmorduc, Alina Pascale, Jihyun An, Han Chu Lee, Hélène Regnault, Dominique Thabut, Giuliana Amaddeo, Massih Ningararhi, Isabelle Olivier-Hourmand, Chloe Metivier, Maxime Ronot, Line Ntandja-Wandji, Violaine Ozenne, Sabrina Sidali, Fabio Marra, Clémence Hollande, Nathalie Ganne-Carrié, Pierre Nahon, Jean-Marie Peron, Marie Lequoy, Rohini Sharma, Paris Liver Cancer Group, Eric Trepo, Sarah Mouri, Manon Allaire, Ju Hyun Shim, Jean-Charles Nault

**Affiliations:** 1 Centre de Recherche des Cordeliers, Sorbonne Université, Inserm, Université de Paris, Team «Functional Genomics of Solid Tumors», Paris, France; 2 Service d'Hépatologie, AP-HP Avicenne, France; 3 Department of Experimental and Clinical Medicine, Internal Medicine and Hepatology Unit, University of Firenze, Florence, Italy; 4 AP-HP, Liver Cancer and Innovative Therapy Unit, Hôpital Beaujon, Clichy, France; 5 Centre de Recherche Sur L'Inflammation (CRI), INSERM, U1149, CNRS, ERL 8252, Paris, France; 6 Nantes Université, CHU Nantes, Institut des Maladies de l’Appareil Digestif (IMAD), Hépato-Gastroentérologie, Inserm CIC 1413, Nantes, France; 7 Centre Hépato-Biliaire, Paul-Brousse Hospital, Assistance Publique-Hôpitaux de Paris, Villejuif, France; 8 Faculté de Médecine, Sorbonne Université and Paris-Saclay Université, Le Kremlin-Bicêtre, France; 9 Unité Mixte de Recherche 1193, Paris-Saclay University, INSERM, Villejuif, France; 10 Gastroenterology and Hepatology, Hanyang University College of Medicine, Guri, South Korea; 11 Gastroenterology, Asan Medical Center, University of Ulsan College of Medicine, Seoul, South Korea; 12 Asan Liver Center, Asan Medical Center, University of Ulsan College of Medicine, Seoul, South Korea; 13 Service d'Hépatologie, AP-HP Henri Mondor, Créteil, France; 14 Hepatogastroenterology Department, La Pitié-Salpêtrière Hospital, AP-HP, Sorbonne Université, Paris, France; 15 INSERM UMR_S 938, Centre de recherche Saint-Antoine, Maladies métaboliques, biliaires et fibro-inflammatoire du foie, Institute of Cardiometabolism and Nutrition (ICAN), Paris, France; 16 CHU Lille, Maladies de l’Appareil Digestif, Université de Lille, Lille, France; 17 Service d'hépato-gastroentérologie, CHU de Caen, ERN RARE-LIVER, France; 18 Department of Radiology, Hôpital Beaujon, AP-HP Nord, Clichy, France; 19 Service d'Hépatologie, AP-HP, Saint-Antoine, France; 20 Unité de Formation et de Recherche Santé Médecine et Biologie Humaine, Université Paris 13, Communauté d’Universités et Etablissements Sorbonne Paris Cité, Paris, France; 21 Service D'hépatologie, Hôpital Rangueil, CHU Toulouse, Toulouse, France; 22 Department of Surgery & Cancer, Imperial College London, Hammersmith Hospital, London, UK; 23 Department of Gastroenterology, Hepatopancreatology and Digestive Oncology, Hôpital Universitaire de Bruxelles, Brussels, Belgium; 24 INSERM UMR 1138, Centre de Recherche des Cordeliers, Genomic Instability, Metabolism, Immunity and Liver Tumorigenesis, Paris, France

**Keywords:** Hepatocellular carcinoma, Immunotherapy, Child-Pugh B, Liver function, ALBI

## Abstract

**Background & Aims:**

We aimed to identify, among patients with advanced hepatocellular carcinoma (HCC) and Child-Pugh B cirrhosis, typically excluded from clinical trials, a subgroup that may benefit from first-line atezolizumab/bevacizumab (A/B).

**Methods:**

We conducted a retrospective international multicenter study including patients with unresectable HCC treated with first-line A/B between 2020 and 2024 across 12 centers. A cohort of Child-Pugh B patients treated with sorafenib served as a control. Baseline clinical, biological, and tumor features were correlated with radiological response, progression-free survival (PFS), and overall survival (OS).

**Results:**

Among 1,499 patients, 246 (16.4%) had Child-Pugh B cirrhosis. Within Child-Pugh B, 72% were B7, 21.5% B8, and 6.5% B9; 73% had albumin–bilirubin (ALBI) grade 2 and 27% grade 3. Median OS and PFS were significantly shorter in Child-Pugh B (8.1 and 5.2 months, respectively) *vs*. Child-Pugh A (16.8 and 8.6 months, respectively; both *p* <0.001). Two-year OS was 20% for Child-Pugh B *vs*. 38% for Child-Pugh A. Child-Pugh B patients treated with A/B had longer OS than those treated with sorafenib (*p* = 0.002). A score combining ALBI grade 1/2 and metastatic status identified prognostic subgroups (10.3 *vs.* 7.9 *vs.* 4.2 months; *p* <0.0001). Improvement to Child-Pugh A occurred in 31% and was associated with recent treatment of underlying liver disease. Radiological response (hazard ratio = 0.58, *p* = 0.021) and liver function improvement (hazard ratio = 0.59, *p* = 0.006) correlated with reduced mortality.

**Conclusions:**

Although Child-Pugh B patients have poorer survival, a subgroup, those with ALBI grade 1/2 and no extrahepatic metastasis, can derive meaningful benefit from A/B therapy. Improving underlying liver disease may contribute to better outcomes.

**Impact and implications:**

Child-Pugh B patients with advanced HCC are systematically underrepresented in clinical trials, creating a critical evidence gap for a population frequently encountered in real-world practice. This large multicenter study shows that a subset of these patients, those with ALBI grade 1/2 and without extrahepatic metastases, can have clinically significant benefit from first-line A/B, providing a practical prognostic tool to guide patient selection. Moreover, the association between treatment of the underlying liver disease and Child-Pugh class improvement suggests that optimizing hepatic function alongside systemic therapy may represent an actionable strategy to improve outcomes, warranting prospective validation.

## Highlights

• Under atezolizumab/bevacizumab, Child-Pugh B patients with HCC (16%) show shorter OS (8.1 months) *vs.* Child-Pugh A (16.8 months).
• Atezolizumab/bevacizumab yields better OS and PFS than sorafenib in Child-Pugh B patients.
• ALBEX score (ALBI grade + metastatic status) stratifies Child-Pugh B into three prognoses groups.
• ALBI grade 1/2 without extrahepatic metastasis identifies patients with potential benefit from atezolizumab/bevacizumab.
• Recent treatment of the etiology of chronic liver disease and disease control at imaging were associated with liver function improvement.

## Graphical abstract

**Figure:**
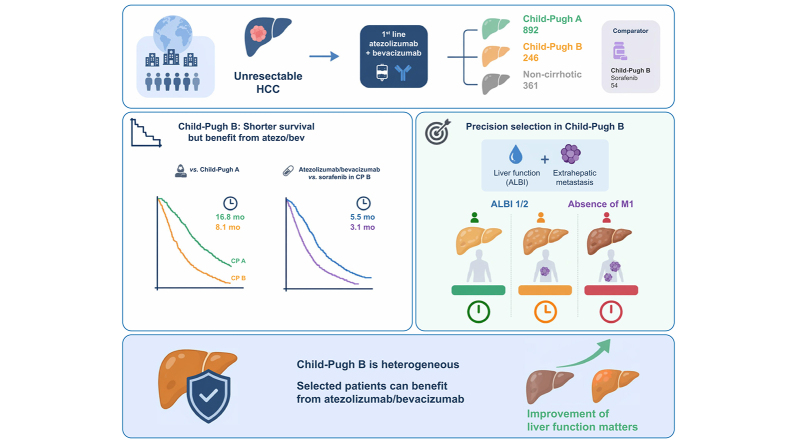

## Introduction

Combination immunotherapies, including atezolizumab/bevacizumab, durvalumab/tremelimumab, and nivolumab/ipilimumab, are now considered standard of care treatments for patients with unresectable hepatocellular carcinoma (HCC), following the demonstration of improved overall survival (OS) in three randomized controlled trials compared with tyrosine kinase inhibitors.[1], [2], [3] Moreover, these combination regimens have shown radiological response rates of ∼25–30%, which are often associated with durable responses.

However, these pivotal trials exclusively enrolled patients with compensated liver function (Child-Pugh class A), as Child-Pugh B status was an exclusion criterion.^4^ Consequently, international guidelines currently recommend systemic therapies only for patients with compensated chronic liver disease, as defined by the Child-Pugh score.^5^^,^^6^ In real-world clinical practice, some patients classified as Child-Pugh B nevertheless receive combination immunotherapy, even though oncological outcomes and safety profiles in this population remain poorly characterized. Notably, Child-Pugh B represents a heterogeneous group, ranging from B7 to B9, and encompassing various albumin–bilirubin (ALBI) grades.^4^^,^^7^ A phase I/II trial evaluating nivolumab monotherapy in Child-Pugh B patients reported an overall response rate of 12%, with a median time to response of 2.7 months and a median duration of response of 9.9 months.^8^ Treatment-related adverse events occurred in 51% of patients, leading to treatment discontinuation in 4%.^8^ Previous retrospective studies have reported reduced OS in Child-Pugh B patients treated with atezolizumab/bevacizumab in clinical practice compared with Child-Pugh A patients, as expected given impaired liver function.[9], [10], [11] However, these studies often involved limited sample sizes, had relatively short follow-up durations or were mainly conducted in Asia.[12], [13], [14], [15], [16] Moreover, these studies lack the granularity needed to stratify Child-Pugh B patients into distinct prognostic subgroups, including incorporating dynamic assessments of liver function and evaluating the impact of treatments for underlying liver disease, analyses that could better inform therapeutic decisions and guide the design of future clinical trials.

The aim of our study is to describe the oncological outcomes of patients with HCC and cirrhosis classified as Child-Pugh B who received first-line systemic treatment with atezolizumab/bevacizumab, and to identify prognostic factors and assess the dynamic evolution of liver function that may help guide the use of combination immunotherapy in this specific patient population.

## Materials and methods

### Inclusion and exclusion criteria of patients with HCC treated by atezolizumab/bevacizumab

The study included patients diagnosed with advanced unresectable HCC and treated with atezolizumab/bevacizumab in a multicenter, real-world cohort of patients treated between July 2020 and December 2024 across 12 tertiary academic centers in France, Italy, UK, and South Korea: Avicenne Hospital (France), Saint-Antoine Hospital (France), Pitié-Salpêtrière Hospital (France), Beaujon Hospital (France), Lille University Hospital (France), Henri Mondor Hospital (France), Caen University Hospital (France), Nantes University Hospital (France), Paul-Brousse Hospital (France), Hammersmith Hospital (UK), Careggi University Hospital (Italy), and Asan Medical Center (South Korea).

The study was conducted in accordance with the ethical guidelines of the Declaration of Helsinki, and the study protocol was approved by local ethics committees (CLEA-2023-no. 309).

The inclusion criteria were the following:

• Diagnosis of HCC based on EASL non-invasive criteria or based on histology with tumor biopsy.^5^
• HCC classified Barcelona Clinic Liver Cancer (BCLC) B progressive under transarterial (chemo) embolization or not accessible to transarterial (chemo) embolization or classified BCLC C.
• Receiving atezolizumab/bevacizumab as a first-line systemic treatment.
• Patients with available clinical, biological, and imaging data at the time of atezolizumab/bevacizumab initiation.

The exclusion criteria were the following:

• Age <18 years.
• Fibrolamellar hepatocellular carcinoma.
• Hepatocholangiocarcinoma.
• Atezolizumab/bevacizumab in second or subsequent line.
• Patients already treated by liver transplantation.
• Atezolizumab/bevacizumab in combination with locoregional therapies (transarterial chemoembolization, selective internal radiotherapy).
• Atezolizumab/bevacizumab as neoadjuvant and/or adjuvant therapy following curative-intent interventions (percutaneous ablation or liver resection).

### Patients with unresectable HCC treated by sorafenib

We also included a cohort of patients with HCC classified as BCLC stage B or C, diagnosed according to EASL guidelines (non-invasive diagnosis or histology), and treated with first-line systemic therapy using sorafenib, a tyrosine kinase inhibitor, between 30 December 2014 and 1 January 2019, in two centers (Erasme Hospital, Brussels, Belgium and Avicenne Hospital, Paris, France).^5^ As only the mRECIST criteria were evaluable in patients treated by sorafenib, we compared the percentages of radiological response in patients treated by sorafenib *vs*. atezolizumab/bevacizumab using mRECIST criteria. Patients were followed-up until 13 October 2021. Among 226 patients treated with sorafenib in this cohort, we selected 54 with Child-Pugh B cirrhosis to compare their oncological outcomes with those of patients with Child-Pugh B cirrhosis receiving atezolizumab/bevacizumab.

### Variables recorded at baseline and during follow-up

The following variables were recorded at baseline, immediately before the initiation of atezolizumab/bevacizumab treatment: age, sex, comorbidities (type 2 diabetes mellitus, arterial hypertension, obesity), BMI, height, weight, etiology of liver disease (HCV, HBV, metabolic syndrome, and past or current chronic alcohol consumption), presence of underlying cirrhosis and its complications (hepatic encephalopathy, ascites, esophageal varices), tumor characteristics (number of nodules, size of the largest nodule, presence of macrovascular invasion, and extrahepatic metastases), BCLC stage, and liver function parameters (Child-Pugh score). Laboratory tests included total bilirubin, prothrombin time, alpha-fetoprotein (AFP), albumin, creatinine, and platelet count.

Data regarding the first radiological evaluation by computed tomography (CT) scan and/or magnetic resonance imaging (MRI) (performed 8–12 weeks after the initiation of treatment) were collected, including tumor response (complete response, partial response, stable disease, or progressive disease), with the response classified according to RECIST 1.1. Patients without any post-baseline radiological tumor assessment were considered as progressor as in the analysis of IMBRAVE150 trial.^1^ Thus, patients were followed-up every 8–12 weeks with clinical examination and laboratory tests including AFP and CT scans and/or MRIs to detect tumor progression.

The following variables were collected at the first radiological evaluation (performed 8–12 weeks after the initiation of treatment) and at the second radiological evaluation (performed 8–12 weeks after the first radiological assessment): Child-Pugh score, ALBI score and serum AFP level.

We also collected data on the management of the underlying liver disease including date of the beginning of antiviral treatment for HBV and subsequent viral suppression viral load, date of the beginning of antiviral treatment for HCV with documentation of a negative HCV PCR at 3 months, and the date of sustained alcohol withdrawal (defined as abstinence maintained at least throughout the period of treatment under atezolizumab/bevacizumab). Successful treatment of the etiology of the underlying liver disease was considered achieved if these conditions were fulfilled before the first dose of atezolizumab/bevacizumab. For each patient, antiviral therapy for HBV and HCV and sustained alcohol withdrawal were harmonized into binary indicators (treated *vs.* not treated), and the interval between effective etiology control and atezolizumab/bevacizumab initiation was calculated. A composite timing variable was then derived, categorizing patients according to the treatment of the etiology of the underlying liver disease: never, at the start of atezolizumab/bevacizumab or ≤1 month before, 1–3 months before the start of atezolizumab/bevacizumab, 3–6 months before the start of atezolizumab/bevacizumab, and >6 months before the start of atezolizumab/bevacizumab.

### Oncological outcomes

The last follow-up date was 1 August 2025. Radiological response was assessed using RECIST 1.1 at the first imaging evaluation and classified as radiological response (complete response + partial response), stable disease, and progressive disease. We also assessed the best radiological response using RECIST1.1. Follow-up data included documentation of radiological progression and death. OS was defined as the time from treatment initiation to death or the last follow-up. Progression-free survival (PFS) was defined as the time from treatment initiation to radiological progression, death, or the last follow-up.

### Statistical analysis

Continuous variables were reported as medians with interquartile ranges, and categorical variables as counts and percentages. Categorical variables were compared using Fisher’s exact test for two groups and the Χ^2^ test for three or more groups. Continuous variables were compared using the non-parametric Mann-Whitney *U* test or Kruskal-Wallis test, according to the number of groups. Survival was analyzed using Kaplan-Meier methods with numbers at risk and the log-rank test. The association between baseline variables and OS and PFS was assessed using univariate Cox regression analysis, and variables with *p* <0.05 were included into multivariate Cox models. Harrel’s C-Index was used to compare the prognostic value (OS) of the different scoring systems. Comparisons of exposure-adjusted incidence rates (EAIRs) between groups were performed using Poisson regression models, with the logarithm of treatment exposure included as an offset.

To investigate the dynamic evolution of liver function during treatment, Child-Pugh class at the first and second imaging assessments was analyzed across categories (A, B, C) and transitions over time were evaluated. Ordered trends across worsening Child-Pugh categories were evaluated using Cochran-Armitage trend tests. To evaluate the time-varying impact of liver function and tumor response, we fitted time-dependent Cox proportional hazards models in a counting-process (start-stop) formulation for OS and PFS, with covariate updates at first and second imaging evaluation. In these models, Child-Pugh class and RECIST response were modeled as time-dependent covariates. Because each patient contributed multiple time intervals, robust standard errors clustered at the patient level were applied to account for within-patient correlation and provide reliable variance estimates. A landmark analysis was also performed to analysis the time-dependent variables. To account for differences in treatment exposure between groups, EAIRs were calculated for adverse events. EAIRs per 100 patient-months were obtained by dividing the number of patients experiencing at least one adverse event by the total cumulative exposure time (in patient-months) during the treatment period, and multiplying by 100. Exposure time was defined as the interval between treatment initiation and treatment discontinuation.

For the comparative effectiveness analysis, we compared atezolizumab/bevacizumab with sorafenib using inverse probability of treatment weighting (IPTW) to account for baseline confounding. A propensity score for receiving atezolizumab/bevacizumab was estimated from baseline variables considered to influence treatment allocation (total bilirubin, size of the largest nodule, more than three nodules, macrovascular invasion, extrahepatic spread, and AFP), and stabilized IPTW for the average treatment effect were derived using several approaches (logistic regression, generalized boosted models, and a Super Learner ensemble). We applied percentile truncation of extreme weights (∼1st and 99th percentiles), selected the method with the lowest mean absolute standardized mean difference across covariates, and assessed covariate balance before and after weighting using standardized mean differences and Love plots. Weighted Kaplan-Meier curves for OS and PFS were subsequently generated using IPTW, and differences between treatment groups were compared using a log-rank test adapted for weighted data (survey-weighted log-rank test).

All analyses were two-sided, with *p* <0.05 considered statistically significant. Statistical analyses were performed using R (R Foundation for Statistical Computing, Vienna, Austria).

## Results

### Description of the population of patients treated with atezolizumab/bevacizumab

**Fig. 1:**
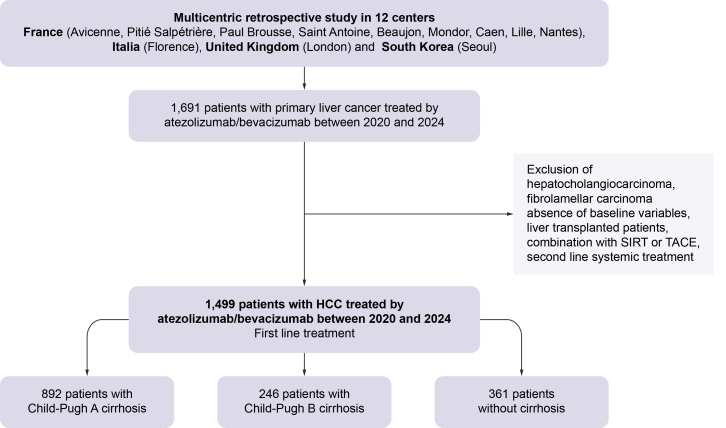
Flow-chart of the study. HCC, hepatocellular carcinoma; SIRT, selective internal radiation therapy; TACE, transarterial chemoembolization.

Among the 1,499 patients with HCC treated by atezolizumab/bevacizumab (Sup Table 1), 1,138 patients (76%) had cirrhosis including 892 patients with Child-Pugh A cirrhosis and 246 patients with Child-Pugh B cirrhosis, whereas 361 patients (24%) did not have cirrhosis (Fig. 1). Patients with Child-Pugh B cirrhosis were younger (*p* <0.001) and more often had alcohol-related cirrhosis (*p* <0.001), larger tumors (*p* <0.001), increased serum AFP (*p* <0.001), higher alkaline phosphatases (*p* <0.001), more portal vein thrombosis (*p* <0.001) and more ALBI grade 3 (*p* <0.001) compared with patients with Child-Pugh A cirrhosis and patients with non-cirrhotic liver (Table 1 and Table S2). Sixty-six Child-Pugh B patients (27% of the whole Child-Pugh B patients) had larges varices. Large varices were not associated with OS (*p* = 0.503), PFS (*p* = 0.428), the occurrence of clinical ascites (*p* = 1.000), or variceal bleeding (*p* = 0.192). In the whole population of 1,499 patients, the median follow-up was 35.7 months. The median OS was 15 months with 34.5% of patients alive at 2 years and 22.4% at 3 years. Early mortality rates were markedly higher in patients with Child-Pugh B cirrhosis compared with those with preserved liver function. 30-, 60-, and 90-day mortality rates were consistently increased in Child-Pugh B patients (6.9%, 15%, and 22%, respectively) compared with Child-Pugh A patients (1.8%, 5.6%, and 8.3%, respectively; all *p* <0.001). The median PFS was 7.4 months with 17% of patients alive without progression at 2 years and 11.5% at 3 years. The first imaging evaluation using RECIST 1.1 criteria identified radiological response in 17.3% of the patients, stable disease in 48.6% of the patients and progressive disease in 34.1% of the patients.

**Table 1:** Description of the population of patients with hepatocellular carcinoma treated with atezolizumab/bevacizumab according to the severity of underlying liver diseases.

Variables	Available data	Cirrhosis Child-Pugh A n = 892	Cirrhosis Child-Pugh B n = 246	Non-cirrhotic n = 361	*p* values∗
Age (years)	1,499	68.06 (13.92)	64.08 (14.07)	69.79 (16.58)	<0.001
Sex (male)	1,499	759/892 (85%)	218/246 (89%)	302/361 (84%)	0.23
Chronic hepatitis B	1,462	174/891 (20%)	76/246 (31%)	88/325 (27%)	<0.001
Chronic hepatitis C	1,462	207/891 (23%)	49/246 (20%)	38/325 (12%)	<0.001
Chronic alcohol intake	1,461	444/890 (50%)	159/246 (65%)	96/325 (30%)	<0.001
Metabolic syndrome	1,462	269/891 (30%)	62/246 (25%)	90/325 (28%)	0.27
Cirrhosis	1,499	892/892 (100%)	246/246 (100%)	0/361 (0%)	<0.001
Size of the index nodule (mm)	1,369	45.00 (55.00)	70.00 (78.00)	68.00 (74.25)	<0.001
Tumor portal vein thrombosis	1,498	328/891 (37%)	132/246 (54%)	106/361 (29%)	<0.001
Extrahepatic metastasis	1,499	299/892 (34%)	93/246 (38%)	171/361 (47%)	<0.001
BCLC stage A	1,498	24/891 (2.7%)	4/246 (1.6%)	13/361 (3.6%)	0.15
BCLC stage B		273/891 (31%)	63/246 (26%)	92/361 (25%)	
BCLC stage C		594/891 (67%)	179/246 (73%)	256/361 (71%)	
Serum AFP (ng/ml)	1,457	54.95 (1,288.13)	210.50 (2,710.85)	74.90 (3,282.80)	<0.001
Platelet count (10^3^/mm^3^)	1,477	148.00 (116.00)	139.00 (111.00)	220.00 (136.50)	<0.001
Total bilirubin (μmol/L)	1,479	13.00 (9.00)	24.15 (24.53)	10.00 (7.40)	<0.001
Albumin (g/L)	1,473	37.00 (7.00)	29.00 (5.00)	36.00 (7.00)	<0.001
INR	1,423	1.10 (1.00–1.20)	1.22 (1.11–1.35)	1.07 (1–1.13)	<0.001
Creatinine (μmol/L)	1,478	72.00 (26.00)	69.00 (27.00)	72.00 (28.77)	0.18
GGT (UI/L)	1,412	170.00 (239.75)	200.00 (248.00)	162.00 (281.50)	0.062
Alkaline Phosphatase (IU/L)	1,441	145.00 (99.00)	194.00 (177.00)	151.00 (152.50)	<0.001
Neutrophiles (/mm^3^)	1,438	3.75 (2.69)	4.60 (4.45)	4.85 (2.82)	<0.001
Clinical ascites	1,493	46/888 (5.2%)	92/246 (37%)	9/359 (2.5%)	<0.001
Overt hepatic encephalopathy	1,493	0 (0%)	0 (0%)	0 (0%)	0.999
ALBI score	1,469	-2.40 (0.64)	-1.56 (0.42)	-2.43 (0.69)	<0.001
ALBI grade 1	1,469	293/870 (34%)	3/246 (1.2%)	133/353 (38%)	<0.001
ALBI grade 2		570/870 (66%)	177/246 (72%)	208/353 (59%)	
ALBI grade 3		7/870 (0.8%)	66/246 (27%)	12/353 (3.4%)	
No esophageal varices	1,272	382/779 (49%)	105/230 (46%)	220/263 (84%)	<0.001
Small esophageal varices		201/779 (26%)	59/230 (26%)	38/263 (14%)	
Large esophageal varices		196/779 (25%)	66/230 (29%)	5/263 (1.9%)	

Data are presented as n (%), n/N (%), or median (IQR).

ALBI, albumin–bilirubin score; BCLC, Barcelona Clinic Liver Cancer; GGT, gamma glutamyl transferase.

∗ Kruskal-Wallis rank sum test; Χ^2^ test; Fisher's exact test.

Next, we analyzed clinical decompensation (occurrence of clinical ascites, over encephalopathy and/or variceal bleeding) in a total of 369 patients for which these data were available (273 patients with Child-Pugh A and 96 with Child-Pugh B). During follow-up, 110 patients (30%) developed clinical decompensation in the absence of radiological progression, with a significantly higher incidence in Child-Pugh B compared with Child-Pugh A (48% *vs.* 23%, *p* <0.001). Among decompensated patients, no significant differences were observed between Child-Pugh score (CPS) groups in the occurrence of acute variceal bleeding (8.4% in Child-Pugh A *vs.* 10% in Child-Pugh B, *p* = 0.556) or hepatic encephalopathy (5.5% in Child-Pugh A *vs.* 9.4% in Child-Pugh, *p* = 0.185). In contrast, occurrence of clinical ascites was significantly more frequent in Child-Pugh B compared with Child-Pugh A (29% *vs.* 14%, *p* = 0.001).

Next, we assessed the frequency of adverse events reported in each group. Differences in safety outcomes, including treatment-related adverse events, between patients with Child-Pugh A and Child-Pugh B cirrhosis are reported in Table S3. Overall, exposure-adjusted incidence rates of adverse events were broadly comparable between the two groups, although fatigue was more frequent in patients with Child-Pugh B cirrhosis. No significant differences were observed between Child-Pugh A and Child-Pugh B patients with respect to grade 3–4 adverse events (Table S4). In patients with CPS A and B, corticosteroid use was 9.1% and 4.3%, respectively, while the use of other immunosuppressants was 5.7% and 4.2% (*p* = 0.592 and *p* = 0.952 respectively).

Treatment duration was significantly shorter in patients with Child-Pugh B compared with Child-Pugh A cirrhosis, with a median duration of 70 days (IQR: 22–221) *vs*. 187 days (IQR: 64–398), respectively (*p* <0.001). Consistently, early treatment discontinuation, defined as treatment duration ≤90 days, was significantly more frequent in patients with Child-Pugh B compared with Child-Pugh A (54% *vs.* 31%, *p*<0.001). Overall, patients with Child-Pugh B cirrhosis had more than a twofold increased likelihood of early treatment discontinuation compared with Child-Pugh A patients (odds ratio 2.56, 95% CI 1.89–3.48). Permanent treatment discontinuation as a result of adverse events was 3.8% in Child-Pugh A patients and 4.9% in Child-Pugh B patients.

### Oncological outcomes in Child-Pugh B patients

Next, we focused on the oncological outcomes of patients with Child-Pugh B cirrhosis compared with patients with Child-Pugh A cirrhosis and patients without cirrhosis. In patients with Child-Pugh B cirrhosis, the median follow-up was 33.8 months. Radiological response at the first imaging evaluation was not statistically different between Child-Pugh A (19%) and Child-Pugh B (14%) whereas more patients with Child-Pugh B (43%) had progressive disease than patients with Child-Pugh A (30%) (*p* = 0.0005). The same results were observed using the best radiological response that was not statistically different in patients with Child-Pugh A (26%) than in Child-Pugh B (18%) whereas more patients with Child-Pugh B (28%) harbored progressive disease than patients with Child-Pugh A (41%) (*p* = 0.0006). The median OS was shorter in Child-Pugh B patients (8.1 months) compared with Child-Pugh A patients (16.9 months) and patients without cirrhosis (15 months, *p* <0.001). In Child-Pugh B patients, the percentages of patients alive at 2 years were 20.4% and 11.7% at 3 years compared with 38.4% at 2 years and 25% at 3 years for Child-Pugh A cirrhosis and 34.5% at 2 years and 23.1% at 3 years for those without cirrhosis. The median PFS was shorter in Child-Pugh B patients (5.2 months) compared with Child-Pugh A patients (8.6 months) and patients without cirrhosis (6.1 months, *p* <0.001). In Child-Pugh B patients, the percentages of patients alive without progression at 2 years were 11.5% and 6% at 3 years compared with 19.7% at 2 years and 12.2% at 3 years for Child-Pugh A cirrhosis and 17.5% at 2 years and 13.1% at 3 years for those without cirrhosis. In Child-Pugh B patients, the median OS was 15.2 months in BCLC B stage *vs*. 6.8 months in BCLC C stage (*p* = 0.0005) and the median PFS was 9.9 months in BCLC B stage *vs*. 3.8 months in BCLC C stage (*p* = 0.003, Fig. S1).

**Fig. 2:**
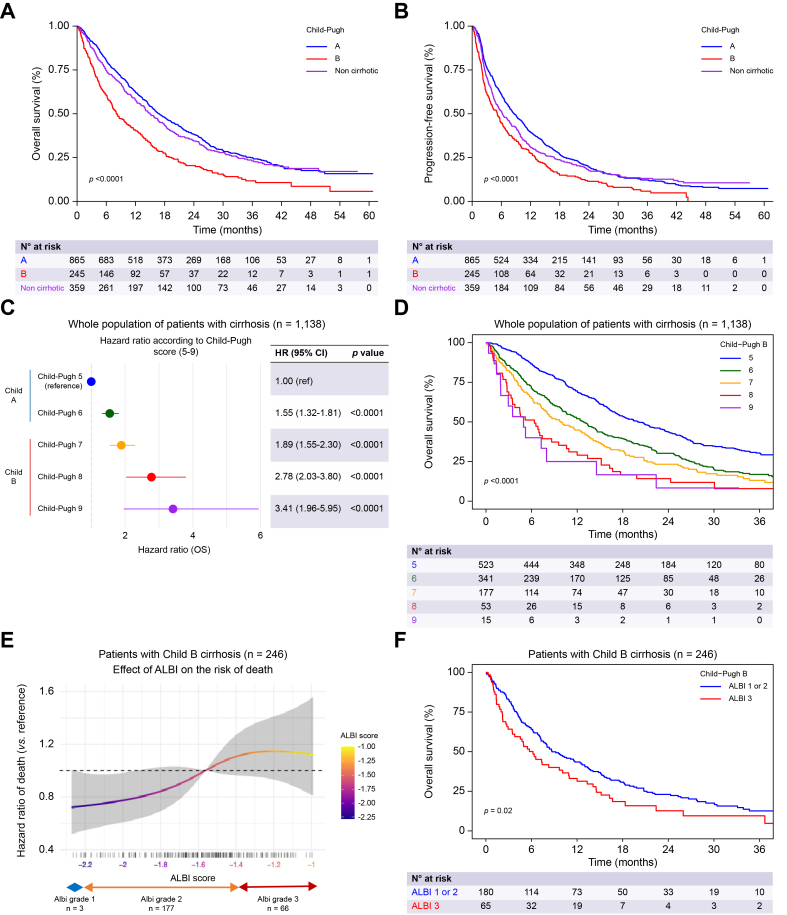
Oncological outcomes of patients with unresectable hepatocellular carcinoma treated with atezolizumab/bevacizumab according to liver function. (A) Kaplan-Meier curve with log-rank test of overall survival in the whole population of 1,499 patients treated with atezolizumab/bevacizumab, stratified by cirrhosis status and Child-Pugh class in patients with cirrhosis. The numbers at risk are represented under the X-axis. (B) Kaplan-Meier curve with log-rank test of progression-free survival in the whole population of 1,499 patients treated with atezolizumab/bevacizumab, stratified by cirrhosis status and Child-Pugh class in patients with cirrhosis. The numbers at risk are represented under the X-axis. (C) Hazard ratio with the 95% confidence interval of overall survival using the Cox model in 1,138 patients with cirrhosis stratified from 5 to 9 using the Child-Pugh score. (D) Kaplan-Meier curve with log-rank test of overall survival in 1,138 patients with cirrhosis treated with atezolizumab/bevacizumab, stratified by Child-Pugh score. The numbers at risk were represented under the X-axis. (E) Association between continuous ALBI score and the risk of death in 246 patients with Child-Pugh B cirrhosis. The plot shows the hazard ratio (HR) for death as a function of the continuous ALBI score, estimated using a Cox proportional hazards model with a restricted cubic spline (4 knots). The solid line represents the estimated HR, and the shaded ribbon the corresponding 95% CI. The horizontal dashed line indicates the null value (HR = 1), corresponding to the reference level of ALBI in the model. The analysis is restricted to the 5–95th percentile range of the ALBI score; tick marks (rug plot) along the X-axis depict the distribution of individual ALBI values in this range. (F) Kaplan-Meier curve with log-rank test of overall survival in 246 patients with Child-Pugh B cirrhosis treated with atezolizumab/bevacizumab, stratified by ALBI score (grade 1/2 *vs.* grade 3). The numbers at risk were represented under the X-axis. ALBI, albumin–bilirubin; HR, hazard ratio.

The Child-Pugh score classified 1,138 patients with cirrhosis in 48% of Child-Pugh A5, 30% of Child-Pugh A6, 16% of Child-Pugh B7, 4% of Child-Pugh B8, and 2% of Child-Pugh B9. Using Cox analysis, the hazard ratio (HR) of the risk of death progressively increased from Child-Pugh A5 (reference) to Child-Pugh B9 (HR = 3.41, 95% CI = 1.96–5.95, *p* <0.001) (Fig. 2C,D). Next, we assessed the prognostic value of ALBI grade in 246 patients with HCC and Child-Pugh B cirrhosis. Three patients (1.2%) were classified as ALBI grade 1 (all these three patients had large volume ascites), 177 patients as ALBI grade 2 (71.9%) and as 66 patients ALBI (26.8%) grade 3. The ALBI score taken as a continuous value was associated with an increasing risk of death (Fig. 2E). The median OS was 8.7 months in patients with Child-Pugh B cirrhosis and ALBI grade 1 or 2 *vs.* 6.2 months in patients with Child-Pugh B cirrhosis and ALBI grade 3 (*p* = 0.02).

### Comparison of Child-Pugh B patients treated with atezolizumab/bevacizumab *vs.* sorafenib

**Fig. 3:**
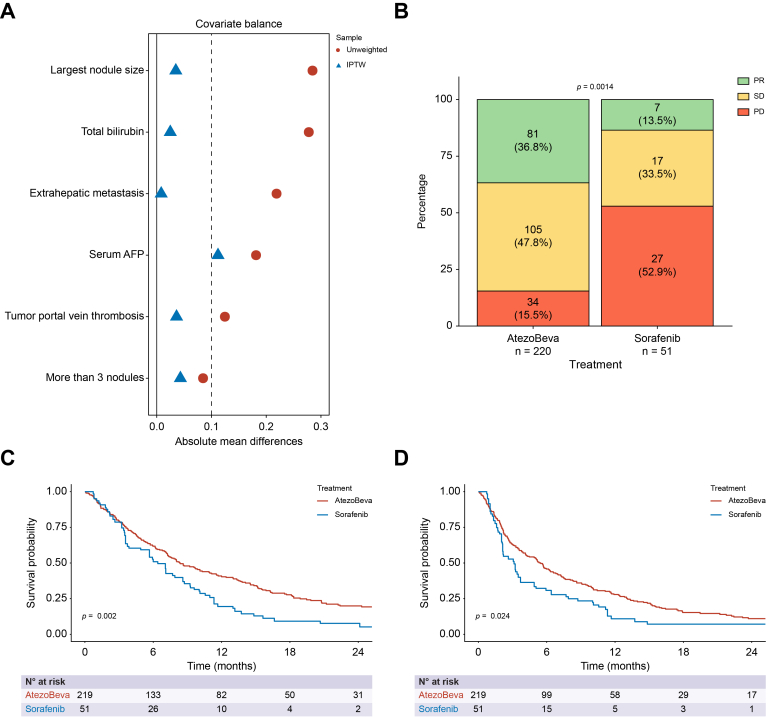
Oncological outcomes of patients with Child-Pugh B cirrhosis and unresectable hepatocellular carcinoma treated by atezolizumab/bevacizumab *vs.* sorafenib after matching using IPTW. (A) Covariate balance before and after inverse probability of treatment weighting (IPTW). Standardized mean differences (SMD) for each baseline covariate in the unweighted sample (red circles) and after IPTW (blue triangles). The vertical dashed line represents the conventional threshold for acceptable balance (SMD <0.10). (B) Distribution of first imaging evaluation using mRECIST 1.1 according to the type of treatments received (sorafenib *vs.* atezolizumab/bevacizumab). The statistical analysis was performed using the Χ^2^ test. (C) Kaplan-Meier curve with log-rank test of overall survival in 219 patients with Child-Pugh B cirrhosis treated with atezolizumab/bevacizumab *vs.* 51 patients with Child-Pugh B cirrhosis treated with sorafenib, after IPTW matching. The numbers at risk were represented under the X-axis. (D) Kaplan-Meier curve with log-rank test of progression-free survival in 219 patients with Child-Pugh B cirrhosis treated with atezolizumab/bevacizumab *vs.* 51 patients with Child-Pugh B cirrhosis treated with sorafenib, after IPTW matching. The numbers at risk were represented under the X-axis.

Next, we aimed to compare the oncological outcomes of patients with Child-Pugh B cirrhosis treated with atezolizumab/bevacizumab in first-line *vs.* those treated with sorafenib in first-line. To this purpose we compared 246 patients with Child-Pugh B cirrhosis among the 1,499 patients treated with atezolizumab/bevacizumab *vs.* 54 patients with Child-Pugh B cirrhosis among the 226 patients treated with sorafenib. Patients with Child-Pugh B cirrhosis treated by sorafenib had less portal vein thrombosis (*p* = 0.049), less extrahepatic metastasis (*p* = 0.001) and an increased total bilirubin level (*p* = 0.025) (Table S2). There was no significant difference in ALBI score between the two groups (*p* = 0.22). We matched the two groups using an IPTW approach taking into account key prognostic variables (tumor size, extrahepatic metastasis, serum AFP, total bilirubin; portal vein thrombosis, number of tumors) (Fig. 3A). After IPTW matching, patients with Child-Pugh B cirrhosis treated with atezolizumab/bevacizumab had a higher radiological response rate than those treated with atezolizumab/bevacizumab using mRECIST criteria (*p* = 0.0014, Fig. 3B). Patients with Child-Pugh B cirrhosis treated with atezolizumab/bevacizumab had longer OS (8.3 months) than those treated with sorafenib (6.4 months; *p* = 0.002; Fig. 3C). Patients with Child-Pugh B cirrhosis treated with atezolizumab/bevacizumab had longer PFS (5.5 months) than those treated with sorafenib (3.1 months; *p* = 0.024; Fig. 3D).

### A simple bio-imaging score predicts oncological outcomes in Child-Pugh B patients

Next, we assessed the baseline variables associated with oncological outcomes. In multivariate analysis, chronic hepatitis C (HR = 0.65, 95% CI = 0.44–0.95, *p* = 0.027), presence of extrahepatic metastasis (HR = 1.49, 95% CI = 1.10–2.03, *p* = 0.011) and ALBI grade 3 (HR = 1.54, 95% CI = 1.10–2.15, *p* = 0.012) were independently associated with the risk of death (Table 2). Moreover, in multivariate analysis, chronic hepatitis C (HR = 0.63, 95% CI = 0.43–0.90, *p* = 0.012), extrahepatic metastasis (HR = 1.60, 95% CI = 1.19–2.14, *p* = 0.002), serum alkaline phosphatase level (HR = 1.00, 95% CI = 1.00–1.00, *p* = 0.043) and Child-Pugh score B8 (HR = 1.44, 95% CI = 1.03–2.03, *p* = 0.035) and B9 (HR = 2.19, 95% CI = 1.20–4.00, *p* = 0.011) were independently associated with PFS (Table 3). We further explored whether the location of extrahepatic metastases influenced prognosis. Data on metastatic sites were available for 912 of 1,138 patients (80.1%), including 725/892 (81.3%) patients with Child-Pugh A and 187/246 (76.0%) with Child-Pugh B cirrhosis. The distribution of metastatic sites did not significantly differ between Child-Pugh A and B patients (all not significant). In the overall cohort, lymph node metastasis was significantly associated with worse OS (HR = 1.36, 95% CI = 1.05–1.77, *p* = 0.020), whereas other metastatic sites (lung, bone, adrenal, central nervous system, and peritoneal) were not significantly associated with outcome. In subgroup analyses, adrenal metastasis was associated with poorer OS in patients with Child-Pugh A cirrhosis (HR = 2.44, 95% CI = 1.01–5.92, *p* = 0.048), whereas in patients with Child-Pugh B cirrhosis, lymph node metastasis remained significantly associated with worse survival (HR = 1.88, 95% CI = 1.08–3.28, *p* = 0.025). Given that detailed information on metastatic sites was only available in a subset of patients, extrahepatic disease was considered as a binary variable (presence *vs.* absence) in subsequent analyses.

**Table 2:** Variables associated with overall survival in patients with Child-Pugh B cirrhosis using Cox analysis.

Variables	Cox univariate analysis for OS			Cox multivariate analysis for OS		
	HR	95% CI	*p* values	HR	95% CI	*p* values
Age (years)	1.00	0.99, 1.02	0.51			
Sex (male)	1.14	0.73, 1.77	0.57			
Chronic hepatitis B	0.95	0.70, 1.29	0.75			
Chronic hepatitis C	0.66	0.46, 0.95	0.025	0.65	0.44, 0.95	0.027
Chronic alcohol intake	1.31	0.97, 1.76	0.076			
Metabolic syndrome	1.32	0.96, 1.80	0.083			
Size of the index nodule (mm)	1.00	1.00, 1.00	0.67			
Tumor portal vein thrombosis	1.43	1.08, 1.90	0.012	0.26	0.93, 1.71	0.137
Extrahepatic metastasis	1.52	1.15, 2.02	0.003	1.49	1.10, 2.03	0.011
Serum AFP (ng/ml)	1.00	1.00, 1.00	0.091			
Platelet count (10^3^/mm^3^)	1.00	1.00, 1.00	0.41			
Total bilirubin (μmol/L)	1.00	1.00, 1.01	0.41			
Albumin (g/L)	0.98	0.94, 1.01	0.15			
INR	0.90	0.64, 1.28	0.56			
Creatinine (μmol/L)	1.00	1.00, 1.01	0.77			
GGT (IU/L)	1.00	1.00, 1.00	0.027	1.00	1.00, 1.00	0.176
Alkaline phosphatase (IU/L)	1.00	1.00, 1.00	0.042	1.00	1.00, 1.00	0.200
Neutrophils (/mm^3^)	1.05	1.00, 1.11	0.044	1.02	0.96, 1.07	0.532
Clinical ascites	1.21	0.91, 1.61	0.19			
No esophageal varices	Ref.	Ref.	Ref.			
Small esophageal varices	1.38	0.97, 1.96	0.074			
Large esophageal varices	1.25	0.88, 1.76	0.21			
ALBI grade 1/2	Ref.	Ref.	Ref.	Ref.	Ref.	Ref.
ALBI grade 3	1.44	1.05, 1.98	0.022	1.54	1.10, 2.15	0.012
Child-Pugh score 7	Ref.	Ref.	Ref.			
Child-Pugh score 8	1.44	1.03, 2.02	0.033			
Child-Pugh score 9	1.68	0.95, 2.98	0.073			

Child-Pugh scores were not analyzed in multivariate analysis because of collinearity with ALBI grade. Statistical analysis was performed using the Cox Model. GGT, gamma glutamyl transferase; HR, hazard ratio; OS, overall survival; Ref., reference.

**Table 3:** Variables associated with progression-free survival in patients with Child-Pugh B cirrhosis using Cox analysis.

Variables	Cox univariate analysis for PFS			Cox multivariate analysis for PFS		
	HR	95% CI	*p* values	HR	95% CI	*p* values
Age (years)	0.99	0.98, 1.01	0.24			
Sex (male)	1.14	0.75, 1.73	0.54			
Chronic hepatitis B	1.14	0.85, 1.51	0.38			
Chronic hepatitis C	0.68	0.48, 0.97	0.031	0.63	0.43, 0.90	0.012
Chronic alcohol intake	1.20	0.90, 1.59	0.21			
Metabolic syndrome	1.17	0.86, 1.58	0.32			
Size of the index nodule (mm)	1.00	1.00, 1.01	0.052			
Tumor portal vein thrombosis	1.34	1.03, 1.75	0.031	1.29	0.97, 1.71	0.085
Extrahepatic metastasis	1.69	1.29, 2.21	<0.001	1.60	1.19, 2.14	0.002
Serum AFP (ng/ml)	1.00	1.00, 1.00	0.24			
Platelet count (10^3^/mm^3^)	1.00	1.00, 1.00	0.14			
Total bilirubin (μmol/L)	1.00	1.0, 1.00	0.97			
Albumin (g/L)	0.97	0.94, 1.00	0.080			
INR	1.00	0.72, 1.38	0.99			
Creatinine (μmol/L)	1.00	1.00, 1.01	0.46			
GGT (IU/L)	1.00	1.00, 1.00	0.083			
Alkaline phosphatase (IU/L)	1.00	1.00, 1.00	0.045	1.00	1.00, 1.00	0.043
Neutrophiles (/mm^3^)	1.06	1.01, 1.11	0.020	1.02	0.97, 1.07	0.460
Clinical ascites	1.24	0.95, 1.63	0.12			
No esophageal varices	Ref.	Ref.	Ref.			
Small esophageal varices	1.50	1.07, 2.11	0.019			
Large esophageal varices	1.30	0.94, 1.81	0.12			
ALBI grade 1/2	Ref.	Ref.	Ref.			
ALBI grade 3	1.31	0.97, 1.76	0.080			
Child-Pugh score 7	Ref.	Ref.	Ref.			
Child-Pugh score 8	1.38	1.00, 1.91	0.054	1.44	1.03, 2.03	0.035
Child-Pugh score 9	1.95	1.13, 3.38	0.017	2.19	1.20, 4.00	0.011

Albumin level, ALBI score and Child-Pugh score were not analyzed in multivariate analysis because of collinearity with ALBI grade. Statistical analysis was performed using the Cox Model. HR, hazard ratio; PFS, progression-free survival; Ref., reference.

**Fig. 4:**
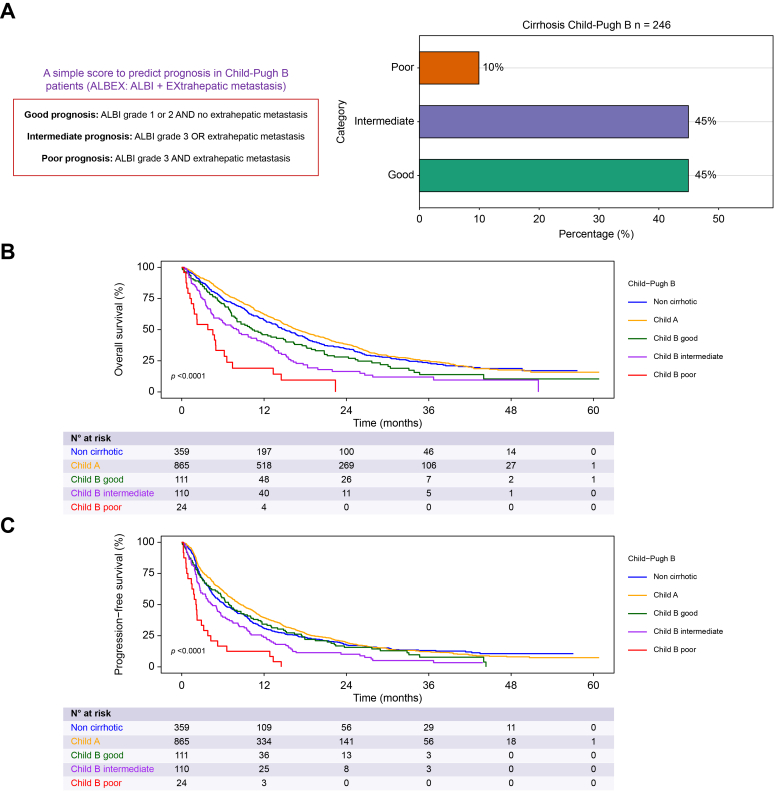
The ALBEX score stratifies patients with Child-Pugh B cirrhosis and unresectable hepatocellular carcinoma treated by atezolizumab/bevacizumab. (A) Definition of the ALBEX score for Child-Pugh B patients combining the ALBI grade with the presence or absence of extrahepatic metastasis in three groups: good prognosis, intermediate prognosis, and poor prognosis. We presented the distribution of the ALBEX score across the three groups among the 246 patients with Child-Pugh B cirrhosis. (B) Kaplan-Meier curve with log-rank test of overall survival in the whole series of 1,499 patients treated with atezolizumab/bevacizumab, stratified by cirrhosis status, Child-Pugh score, and, for Child-Pugh B patients, ALBEX score (good, intermediate, or poor prognosis). The numbers at risk were represented under the X-axis. (C) Kaplan-Meier curve with log-rank test of progression-free survival in the whole series of 1,499 patients treated with atezolizumab/bevacizumab, stratified by cirrhosis status, Child-Pugh score, and, for Child-Pugh B patients, ALBEX score (good, intermediate, or poor prognosis). The numbers at risk were represented under the X-axis.

We constructed a simple bio-imaging score to predict oncological outcomes based on the results of multivariate analysis of OS. We divided patients with Child-Pugh B cirrhosis into three groups: good prognosis with patients with ALBI grade 1 or 2 AND absence of extrahepatic metastasis, intermediate prognosis with patients with ALBI grade 3 OR absence of extrahepatic metastasis, and poor prognosis with patients with ALBI grade 3 AND absence of extrahepatic metastasis (the ALBEX score, Fig. 4A). The distribution of good, intermediate, and poor prognosis in Child-Pugh B patients was 40%, 40%, and 10% respectively (Fig. 4A). The median OS was 16.9 months in Child-Pugh A, 15 months in non-cirrhotic liver, 10.3 months in Child-Pugh B of good prognosis, 7.9 months in Child-Pugh B of intermediate prognosis, and 4.2 months in Child-Pugh B of poor prognosis (*p* <0.0001, Fig. 4B). The same results were observed for PFS (*p* <0.0001, Fig. 4C). At 1-year follow-up, all patients classified in the Child-Pugh B poor-prognosis group had either died or shown disease progression, and by 2 years all had died. The ALBEX (ALBI + extrahepatic metastasis) score showed a modest improvement in discrimination compared with ALBI and Child-Pugh subclassification, with a higher C-Index observed for ALBEX (0.58) than for ALBI (0.54) and CPS (0.55) to predict OS (Fig. S2).

### Variation of liver function, role of treatment of underlying liver disease, and impact on OS

**Fig. 5:**
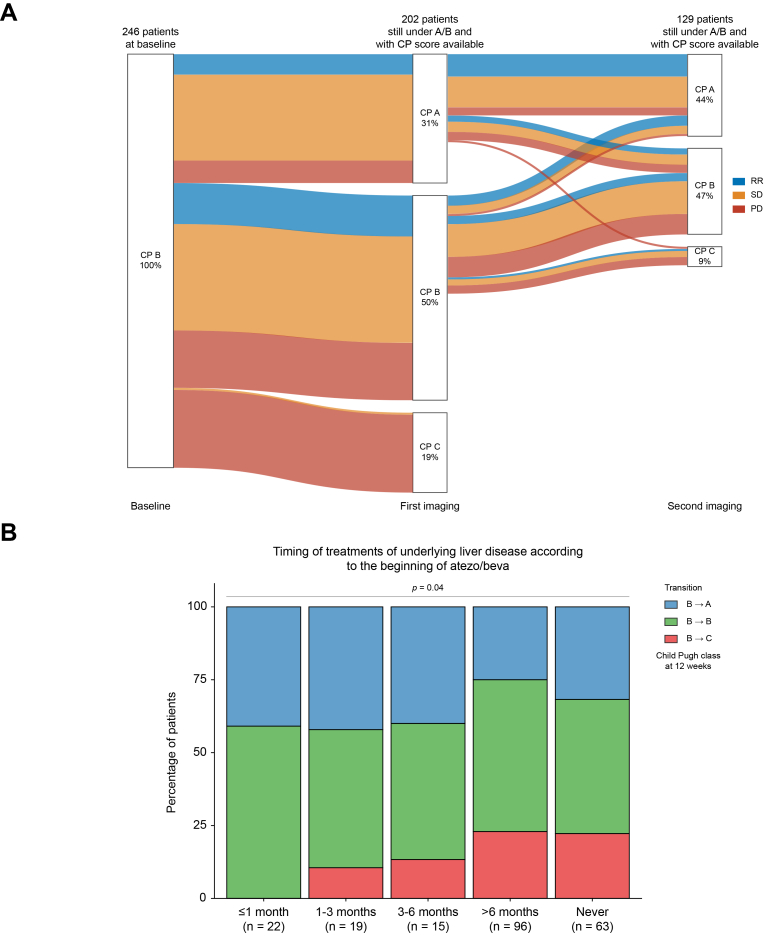
Evolution of liver function in patients with Child-Pugh B cirrhosis and unresectable hepatocellular carcinoma treated by atezolizumab/bevacizumab. (A) Evolution of Child-Pugh class and treatment response in 246 patients with Child-Pugh B cirrhosis at baseline and with hepatocellular carcinoma receiving atezolizumab/bevacizumab. This alluvial plot shows transitions in Child-Pugh class (CP) from baseline to the time of the first (10–12 weeks) and second (20–24 weeks) radiological evaluations after the initiation of systemic treatment. Flow width is proportional to the number of patients moving between Child-Pugh classes. Colors represent tumor response categories (RR = radiological response, SD = stable disease, PD = progressive disease). Percentages indicate the distribution of Child-Pugh classes at each time point. (B) Timing of the treatments of the etiologies of chronic liver disease and subsequent Child-Pugh transitions in 246 patients initially classified as Child-Pugh B. This stacked bar plot displays the evolution of liver function (Child-Pugh score) at the first imaging evaluation (10–12 weeks after the beginning of the systemic treatment) according to the timing of treatments targeting the underlying etiologies of chronic liver disease (*e.g.* antiviral therapy, alcohol withdrawal) relative to the start of atezolizumab/bevacizumab. Child-Pugh transitions are categorized as improvement (B→A, blue), stability (B→B, green), or deterioration (B→C, red). Each bar represents the percentage of patients within a given timing group (never, at the start of atezolizumab/bevacizumab or ≤1 month before, 1–3 months before the start of atezolizumab/bevacizumab, 3–6 months before the start of atezolizumab/bevacizumab, and >6 months before the start of atezolizumab/bevacizumab). Statistical analysis was performed using the Cochran-Armitage trend test.

Next, we evaluated changes in the Child-Pugh score from baseline to the first radiological assessment (10–12 weeks after initiation of systemic treatment) in 202 patients who were still receiving atezolizumab/bevacizumab and had available Child-Pugh data. We then assessed changes to the second radiological evaluation (20–24 weeks after treatment initiation) in 129 patients who were still on atezolizumab/bevacizumab and for whom Child-Pugh scores were available. At the first radiological evaluation, 31% of the patients were classified as Child-Pugh A, 50% as Child-Pugh B, and 19% as Child-Pugh C (Fig. 5A). At the second radiological evaluation, 44% of the patients were classified as Child-Pugh A, 47% as Child-Pugh B, and 9% as Child-Pugh C (Fig. 5A). Progressive disease was associated with a higher rate of progression from Child-Pugh B to C at the first (Cochrane-Armitage trend test, *p* <0.001) and second (Cochrane-Armitage trend test, *p* <0.001) imaging evaluations. Child-Pugh class was reassessed at the time of the first radiological evaluation, allowing liver function evolution and tumor response to be evaluated concurrently. Among baseline Child-Pugh B patients, 63 out of 202 (31.2%) improved to Child-Pugh A, 100 (49.5%) remained Child-Pugh B, and 39 (19.3%) worsened to Child-Pugh C. When analyzing the full distribution of radiological response across Child-Pugh transitions, a significant association was observed (*p* <0.001), mainly driven by a higher proportion of progressive disease among patients who worsened to Child-Pugh C. However, when specifically focusing on improvement from Child-Pugh B to A, no significant association was observed with radiological response (*p* = 0.832). Interestingly, a small subset of patients with progressive disease at first radiological evaluation nevertheless improved from Child-Pugh B to A. Among these patients (n = 11), the majority had received treatment for the underlying liver disease. Among patients with viral hepatitis, six of seven (85.7%) with HBV and one (100%) with HCV were treated, whereas alcohol cessation was reported in four of six (66.7%) of patients with alcohol-related liver disease. To account for potential survivor bias, we performed a 12-week landmark analysis including only patients alive and evaluable at the time of the first radiological assessment. In this analysis, improvement from Child-Pugh B to A remained associated with longer OS compared with patients who remained Child-Pugh B or worsened to Child-Pugh C (Fig. S3). Median OS from the landmark time was 13.6 months for patients improving to Child-Pugh A, 9.7 months for those remaining Child-Pugh B, and 4.3 months for those worsening to Child-Pugh C. In univariate Cox analysis, remaining Child-Pugh B (HR = 1.48, 95% CI = 1.03–2.12, *p* = 0.033) and worsening to Child-Pugh C (HR = 2.29, 95% CI = 1.19–4.43, *p* = 0.013) were associated with an increased risk of death compared with improvement to Child-Pugh A.

We also evaluated the impact of treatment for the underlying liver disease on changes in the Child-Pugh score between baseline and the first radiological assessment. Patients were stratified according to the timing of liver disease treatment relative to the initiation of systemic therapy: never treated; treated at the start of atezolizumab/bevacizumab or ≤1 month before; 1–3 months before; 3–6 months before; and >6 months before the start of atezolizumab/bevacizumab. Patients who received recent treatment showed greater improvement in liver function at the first evaluation compared with those treated >6 months earlier or never treated (*p* = 0.04, Cochran-Armitage trend test, Fig. 5B). Among patients with Child-Pugh B cirrhosis at baseline, liver-directed interventions were common. Antiviral therapy was administered in 81.6% of patients who were HBV-positive and 61.2% of patients who were HCV-positive, whereas 78.6% of patients with alcohol-related liver disease reported abstinence. No significant association was observed between these interventions and improvement in liver function at first evaluation (HBV *p* = 0.176; HCV *p* = 0.114; alcohol *p* = 1.000). Exploratory analyses evaluating the timing of these interventions relative to treatment initiation did not demonstrate a significant overall association. However, a modest trend suggested that patients receiving very recent interventions (≤1 month) may more frequently experience improvement in liver function. Among patients with Child-Pugh B cirrhosis with available data on liver-directed interventions, antiviral therapy achieved control of the etiology in 81% of the patients who were HBV-positive and 61% of the patients who were HCV-positive, whereas 72% of patients with alcohol-related liver disease reported abstinence at baseline or during a part of the follow-up. No significant association was observed between these interventions and improvement in liver function at first evaluation (HBV *p* = 0.176; HCV *p* = 0.114; alcohol *p* = 0.80). Exploratory analyses evaluating the timing of these interventions relative to treatment initiation did not demonstrate a significant overall association but these analyses were limited by the small number of patients analyzed in each subgroup.

Finally, we performed a time-dependent Cox analysis to assess the impact of radiological response and liver function improvement on OS. Patients whose Child-Pugh class improved from B to A had a significantly lower risk of death compared with those who remained in class B (HR = 0.59, 95% CI = 0.40–0.86, *p* = 0.006). Conversely, patients with progressive disease had more than a twofold higher risk of death (HR = 2.38, 95% CI = 1.42–4.00, *p* = 0.001).

## Discussion

Herein, we present the results from a large, multicenter cohort spanning several countries, including Asian centers, with diverse etiologies and a homogeneous treatment regimen (atezolizumab/bevacizumab). Patients were stratified according to the presence or absence of cirrhosis and by degree of liver dysfunction. Previous cohorts evaluating immunotherapy in Child-Pugh B patients have reported a median OS of ∼6 months,[9], [10], [11] but these studies were heterogeneous in terms of patient populations, treatments received, and were limited by short follow-up or were mainly conducted in Asia.[12], [13], [14], [15], [16] In contrast, our study benefits from a long-term follow-up of about 35 months. We observed a median OS of 8 months, with 20.4% of patients alive at 2 years and 11.7% at 3 years for patients with Child-Pugh B cirrhosis treated with atezolizumab/bevacizumab. In the observational GIDEON study, the median OS of Child-Pugh B patients treated with sorafenib was 5.2 months.^17^ In our study, after matching, comparison with a cohort treated with sorafenib showed longer OS and PFS as well as better radiological response in the atezolizumab/bevacizumab group, suggesting superior oncologic efficacy even among Child-Pugh B patients.^18^

However, it was difficult to define a futility cut-off based on these non-stratified data, although some patients, particularly those with Child-Pugh B7 or ALBI grade 2, had a longer survival. In contrast, patients with ALBI grade 3 or Child-Pugh B8/B9 had, as expected, shorter OS and PFS. We attempted to identify a futility group by combining ALBI grade 3 with the presence of extrahepatic metastasis and found that this small subgroup of Child-Pugh B patients uniformly died before 2 years and had either died or progressed by 1 year. Interestingly, extrahepatic metastases have previously been associated with higher rates of progressive disease or poorest OS in patients with advanced HCC treated with atezolizumab/bevacizumab.^19^^,^^20^ Conversely, a good-prognosis subgroup, those with ALBI grade 2 and no extrahepatic metastasis, appears to be a reasonable population to treat with atezolizumab/bevacizumab.

Moreover, liver function should be considered a dynamic rather than a static parameter, as one-third of patients demonstrated improvement at the first radiological evaluation. In contrast, most patients who experienced disease progression showed a deterioration in liver function, worsening from Child-Pugh B to C. We also assessed the impact of treating the underlying liver disease (antiviral therapy, alcohol withdrawal) on changes in liver function from baseline to the first imaging assessment. However, subgroup analyses stratified by etiological treatment type should be interpreted with caution, given the limited sample size and the resulting lack of statistical power. Furthermore, the retrospective design of the cohort may have introduced assessment bias regarding alcohol withdrawal, and fluctuations in alcohol abstinence during follow-up could not be rigorously evaluated. Recent treatment of the underlying liver disease (within 1–3 months) was associated with greater improvement in liver function compared with patients who had not received such treatment or who had been treated more than 6 months earlier. Patients who recently received treatment for their underlying liver disease were more likely to experience improved liver function than those treated further in the past, suggesting that, in these latter patients, liver impairment is more likely driven by the tumor itself. These findings support the potential benefit of managing underlying liver disease in Child-Pugh B patients, consistent with previous studies demonstrating positive effects in compensated cirrhosis.[21], [22], [23]

We also showed that both liver function and response to atezolizumab/bevacizumab evolve dynamically and influence patient prognosis. Radiological response and improvement in liver function were each independently associated with better OS, suggesting that effective treatment of the underlying liver disease, combined with a potent antitumor response, may be beneficial for Child-Pugh B patients.

Limitations of our study include the retrospective design, clinicians' selection bias toward Child-Pugh B patients with better prognosis, and the lack of granular data on adverse events. In addition, detailed data on bevacizumab dose intensity, including dose reductions and treatment modifications over time, were not systematically collected, precluding a dedicated analysis of treatment exposure. However, we previously showed that adverse events are difficult to evaluate in retrospective studies.^24^ In addition, the comparison between atezolizumab/bevacizumab and sorafenib was based on a non-randomized analysis using a historical control cohort, which may introduce residual confounding despite IPTW adjustment. Some important prognostic variables, such as ECOG performance status, frailty, model for end-stage liver disease score, and detailed portal hypertension, were not consistently available and could not be included in the model. Therefore, confounding by indication cannot be excluded, and these findings should be considered hypothesis-generating rather than demonstrating a causal effect. Consequently, ongoing prospective non-randomized trials (SIERRA trial NCT05883644, HESTIA trial NCT05622071) testing combo-immunotherapy in Child-Pugh B patients will be valuable for better assessing safety and signal of antitumor efficacy. Moreover, the optimal immunotherapy regimen for patients with CP-B cirrhosis remains to be assess.

In conclusion, patients with HCC and Child-Pugh B cirrhosis are associated with shorter OS under atezolizumab/bevacizumab; however, a subset of patients demonstrated meaningful survival, with 20.4% alive at 2 years. The combination of ALBI grade 3 and extrahepatic metastasis identifies a subgroup with a very poor prognosis in whom treatment appears to be futile. Treatment of the etiology of underlying liver disease may help improve OS, particularly when combined with achieving disease control with immunotherapy.

## Abbreviations

AFP, alpha-fetoprotein; ALBI, albumin–bilirubin; BCLC, Barcelona Clinic Liver Cancer; CPS, Child-Pugh score; CT, computed tomography; EAIRs, exposure-adjusted incidence rates; GGT, gamma glutamyl transferase; HCC, hepatocellular carcinoma; HR, hazard ratio; IPTW, inverse probability of treatment weighting; MRI, magnetic resonance imaging; OS, overall survival; PFS, progression-free survival; SIRT, selective internal radiation therapy; TACE, transarterial chemoembolization.

## Authors’ contributions

No medical writer or editor was involved in the development of this manuscript. Conceptualized the study: AVO, CC, JCN. Generated the data: AVO, CC, MB, YT, VD, OR, AP, JA, HCL, HR, DT, GA, MN, IOH, CM, MR, LNW, VO, SS, FM, CH, NGC, PN, JMP, ML, RS, MA, ET, JHS, JCN. Managed the data curation: AVO, CC, JCN. Conducted the formal analysis: AVO, CC, JCN. Wrote the original draft: AVO, CC, JCN. Reviewed and edited the final draft: all authors. Access to the study data and final responsibility for the submission of the manuscript: all authors.

## Data availability

The data supporting the study findings, as well as additional are available from the corresponding author (JCN) upon reasonable request from publication date.

## Financial support

No financial support was received to produce this manuscript.

## Conflicts of interest

JCN received research grants from Bayer and Ipsen. YT had COI with Roche. MA reports receiving consulting fees from AstraZeneca and Roche, and speaker honoraria from AstraZeneca, Bayer, AbbVie, Gilead, Roche, and Sirtex. MR served as consultant for Bayer and Sirtex, and received speakers fees from Canon, Guerbet, Terumo and Sirtex. MN reports receiving consulting fees from AstraZeneca, and speaker honoraria from AstraZeneca, Roche, and Chiesi. HR reports receiving consulting fees from Boston and Sirtex. IOH has received honoraria from and/or travel expenses from AstraZeneca, Roche, and AbbVie. MB declares consulting fees Bayer, MSD, Sirtex Medical, and Roche; advisory board fees from Bayer, MSD, Sirtex Medical, Eisai, AstraZeneca, Ipsen, Servier, Taiho, BMS, and Terumo, payment or honoraria for lectures from Bayer, Roche; MSD, Sirtex Medical, and AstraZeneca. OR received honoraria from Roche for lectures and support for attending meetings or travel. NGC received travel and congress fees, Consulting fees or honoraria for lectures, presentations, speakers bureaus from AbbVie, Gilead, and Roche. The remaining authors declare no conflicts of interest that pertain to this work.

Please refer to the accompanying ICMJE disclosure forms for further details.
