# Supplementary material for: Clinical predictors of response to atezolizumab/bevacizumab in Child-Pugh B patients with hepatocellular carcinoma
Source: JHEP Rep. 2026 Jun 11;8(9):101915. doi: 10.1016/j.jhepr.2026.101915 (PMC13453049; doi:10.1016/j.jhepr.2026.101915)
Supplement: Supplementary file 1 [file mmc1.pdf]

Clinical predictors of response to atezolizumab/bevacizumab in Child-Pugh B patients with hepatocellular carcinoma

Anne Victoire Odent, Claudia Campani, Mohamed Bouattour, Yann Touchefeu, Victoria Delhoume, Olivier Rosmorduc, Alina Pascale, Jihyun An, Han Chu Lee, Hélène Regnault, Dominique Thabut, Giuliana Amaddeo, Massih Ningararhi, Isabelle Olivier-Hourmand, Chloe Metivier, Maxime Ronot, Line Ntandja-Wandji, Violaine Ozenne, Sabrina Sidali, Fabio Marra, Clémence Hollande, Nathalie Ganne-Carrié, Pierre Nahon, Jean-Marie Peron, Marie Lequoy, Rohini Sharma, Paris Liver Cancer Group, Eric Trepo, Sarah Mouri, Manon Allaire, Ju Hyun Shim, Jean-Charles Nault

Table of contents

Fig. S1……..2

Fig. S2……..3

Fig. S3……..4

Table S1……...5

Table S2……...6

Table S3……...7

Table S4……..8

Fig. S1: Oncological outcomes of patients with Child Pugh B cirrhosis and unresectabl e HCC treated by atezolizumab/bevacizumab stratified according to BCLC stage

A) Kaplan Meier curve with log rank test of overall survival in the patients with Child Pugh B cirrhosis treated by atezolizumab/bevacizumab and stratified in BCLC B stage versus BCLC C stage. The numbers at risk were represented under the X axis.
B) Kaplan Meier curve with log rank test of progression-free survival in the patients with Child Pugh B cirrhosis treated by atezolizumab/bevacizumab and stratified in BCLC B stage versus BCLC C stage. The numbers at risk were represented under the X axis.

Fig. S1

A

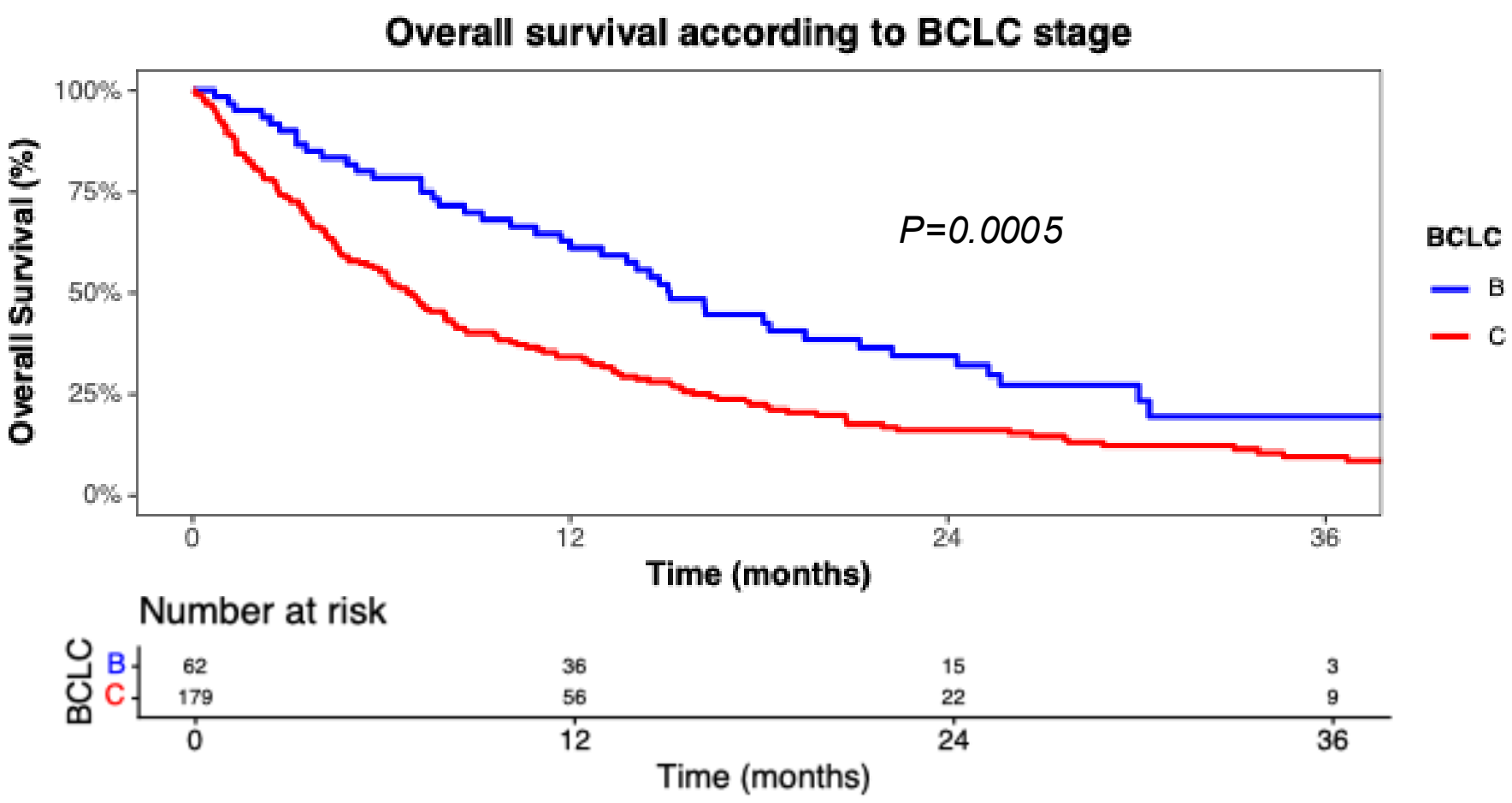

B

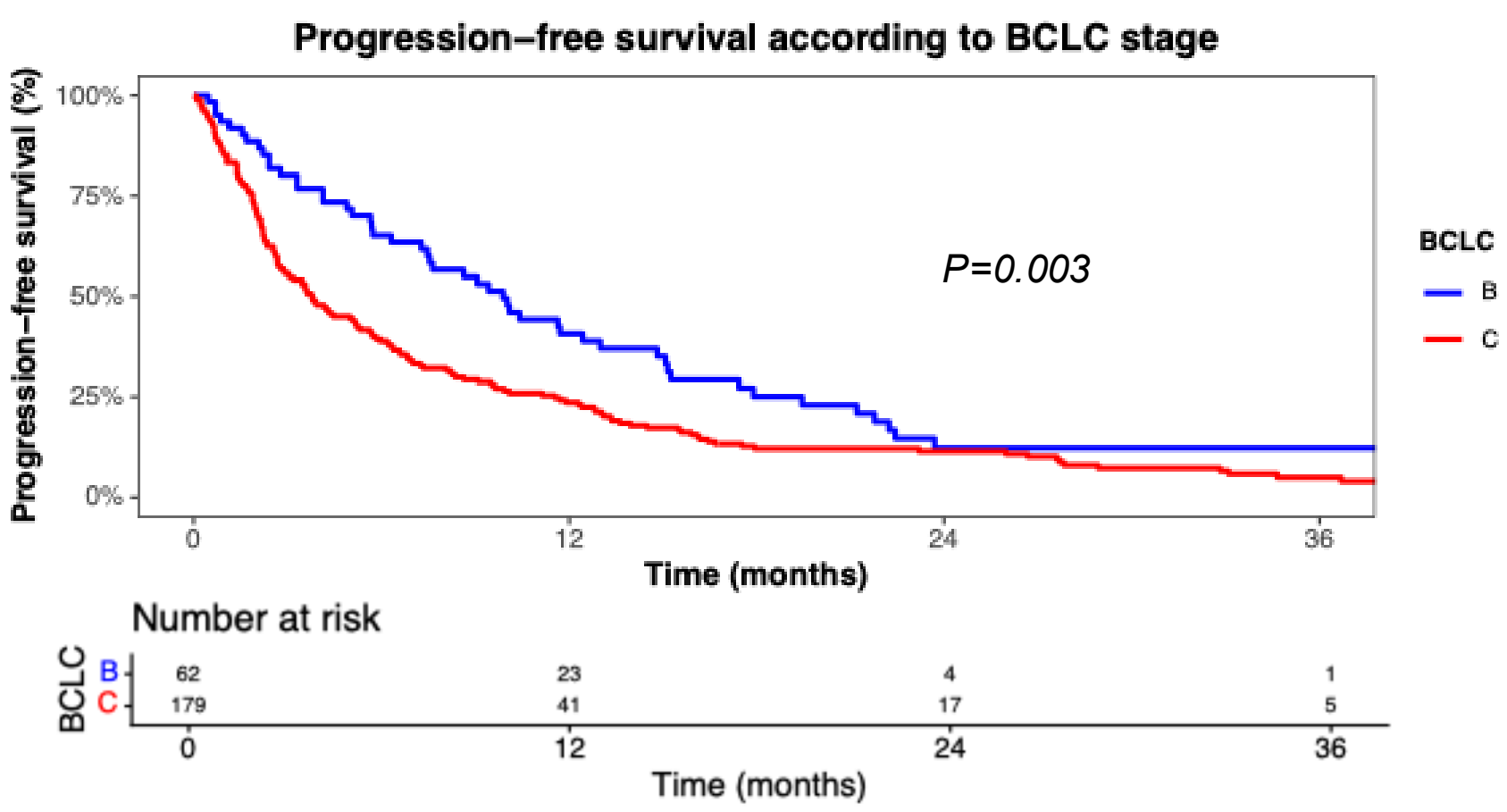

Fig. S2: c-Harrel index of ALBEX score, Child Pugh Score and ALBI score for the prediction of overall survival in Child Pugh B

CPS= Child Pugh Score, ALBI: albumin Bilirubin score. We used the c-Harrel index for statistical analysis.

Fig. S2

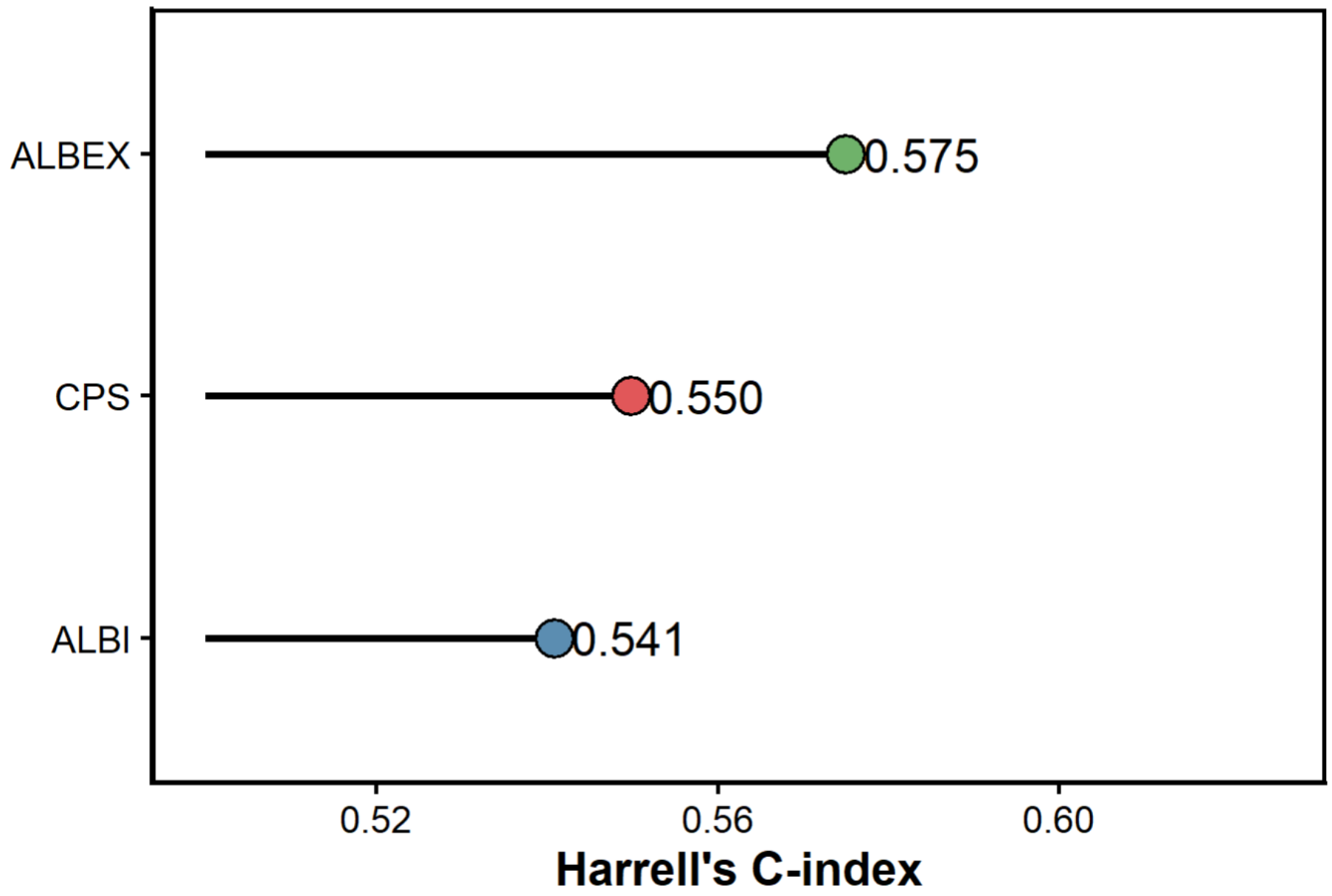

Fig. S3: Impact of Child-Pugh Score Modification on Overall Survival: A 12- Week Landmark Analysis

B -> A = improvement from Child Pugh B (baseline) to Chilg Pugh A (12 weeks), B -> B = stability from Child Pugh B (baseline) to Chilg Pugh A (12 weeks), B -> C = degradation from Child Pugh B (baseline) to Chilg Pugh A (12 weeks). A landmark analysis was performed to analysis these time dependent variables and we represented the results using the Kaplan-Meier Method with the log-rank test.

Fig. S3

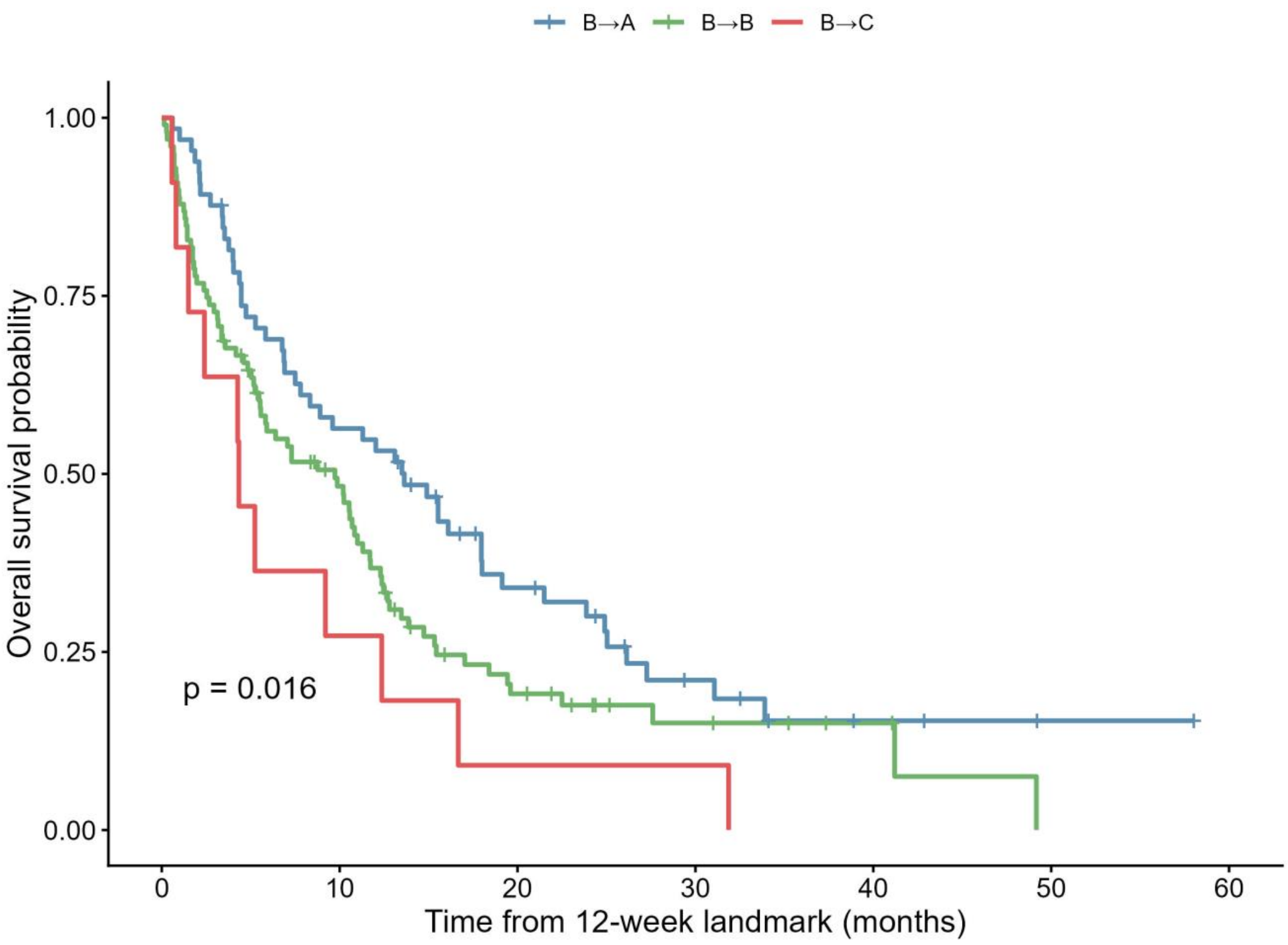

Table S1: description of the whole population of 1499 patients with HCC treated by atezolizumab /bevacizumab

Variables	Available data	Numbers (percentages) Median (IQR) N = 1499
Age (years old)	1499	68.00 (15.00)
Gender (Male)	1499	1,279 (85%)
Chronic hepatitis B	1,462	338 (23%)
Chronic hepatitis C	1,462	294 (20%)
Chronic alcohol intake	1,461	699 (48%)
Metabolic syndrome	1,462	421 (29%)
Cirrhosis	1,499	1138 (76%)
Size of the index nodule (mm)	1,369	55.00 (67.00)
Tumor portal vein thrombosis	1,498	566 (38%)
Extrahepatic metastasis	1,499	563 (38%)
BCLC stage A	1,498	41 (2.7%)
BCLC stage B		428 (29%)
BCLC stage C		1,029 (69%)
Serum AFP (ng/ml)	1,457	72.80 (1,973.30)
Platelet count ($10^3/mm^3$)	1,477	164.00 (133.00)
Total Bilirubin (µmol/l)	1,479	13.00 (11.15)
Albumin (g/l)	1,473	36.00 (7.00)
Creatinin (µmol/l)	1,478	72.00 (28.00)
Gamma glutamyl transferase (UI/L)	1,412	174.00 (251.00)
Alkaline Phosphatase (UI/l)	1,441	154.00 (123.00)
Neutrophiles ($/mm^3$)	1,438	4.10 (3.05)
Clinical ascites	1493	146 (9.8%)
ALBI score	1,469	-2.31 (0.79)
Child Pugh A cirrhosis	1138*	892 (78.3%)
Child Pugh B cirrhosis	1138*	246 (21.7%)
ALBI grade 1	1,469	429 (29%)
ALBI grade 2		955 (65%)
ALBI grade 3		85 (5.8%)
No Esophageal varices	1,272	707 (56%)
Small Esophageal varices		298 (23%)
Large Esophageal varices		267 (21%)

[2]Kruskal-Wallis rank sum test; Chi-squared test; Fisher's exact test * cirrhosis only. ALBI = albumin bilirubin score BCLC = Barcelona Clinic Liver Cancer

Table S2: comparison of patients with Child Pugh B cirrhosis treated by atezolizumab/bevacizumab versus patients treated by sorafenib before IPTW matching

Variables	Available data	AtezoBeva N = 246[1]	Sorafenib N = 54[1]	P-value[2]
**Age (years old)**	300	64.08 (56.90, 71.00)	64.69 (56.41, 71.43)	0.741
**Gender (Male)**	300	218 (89%)	47 (87%)	0.743
**Chronic hepatitis B**	300	76 (31%)	13 (24%)	0.320
**Chronic hepatitis C**	300	49 (20%)	12 (22%)	0.703
**Chronic alcohol intake**	300	159 (65%)	33 (61%)	0.625
**Metabolic syndrome**	300	62 (25%)	9 (17%)	0.181
**Cirrhosis**	300	246 (100%)	54 (100%)	1
**Size of the index nodule (mm)**	278	70.00 (32.00, 110.00)	60.00 (31.00, 98.00)	0.209
**Tumor portal vein thrombosis**	300	132 (54%)	21 (39%)	**0.049**
**Extrahepatic metastasis**	300	93 (38%)	8 (15%)	**0.001**
**BCLC stage A**	300	4 (1.6%)	2 (3.7%)	**0.007**
**BCLC stage B**		63 (26%)	24 (44%)	
**BCLC stage C**		179 (73%)	28 (52%)	
**Serum AFP (ng/ml)**	295	210.50 (21.90, 2,751.50)	122.00 (9.60, 954.00)	0.158
**Platelet count ($10^3/mm^3$)**	299	139.00 (98.00, 209.00)	124.50 (80.00, 199.00)	0.362
**Total Bilirubin (µmol/l)**	300	24.15 (14.00, 38.70)	34.20 (18.00, 42.00)	**0.025**
**Albumin (g/l)**	299	29.00 (27.00, 32.00)	29.00 (26.00, 33.00)	0.833
**Creatinin (µmol/l)**	299	69.00 (58.00, 85.00)	74.00 (63.00, 82.00)	0.208
**Clinical ascites**	300	92 (37%)	16 (30%)	0.281
**ALBI grade 1**	299	3 (1.2%)	0 (0%)	0.22
**ALBI grade 2**		179 (73%)	33 (62%)	
**ALBI grade 3**		64 (26%)	20 (38%)	

[1]Number(%), median (interquartile range) [2]Wilcoxon rank sum test; Pearson's Chi-squared test; Fisher's exact test

AFP: alpha foetoprotein, ALBI: albumin bilirubin, BCLC = Barcelona Clinic Liver Cancer

Table S3: Exposure-adjusted incidence rates of all grade adverse events in Child-Pugh A and B patients

Adverse event	Child-Pugh A	Child-Pugh B	p-value
Diarrhoea/colitis	0.50	0.71	0.519
Fatigue	3.68	6.66	**0.001**
Hepatitis	0.36	0.38	0.961
Arterial hypertension	1.60	1.03	0.262
Hyperthyroidism	0.94	1.13	0.675
Hypophysitis	0.00	0.00	0.999
Hypothyroidism	1.44	1.47	0.953
Mucositis	0.19	0.00	0.994
Palmar-plantar erythrodysaesthesia	0.30	0.19	0.651
Pneumonitis	0.24	0.61	0.264
Proteinuria	0.96	0.59	0.349
Pruritus	1.06	1.53	0.460
Rash	1.20	1.61	0.371

Exposure-adjusted incidence rates (EAIRs), expressed per 100 patient-months, were calculated by dividing the number of patients experiencing at least one adverse event by the total cumulative exposure time from treatment initiation to discontinuation, multiplied by 100. Comparisons of exposure-adjusted incidence rates (EAIRs) between groups were performed using Poisson regression models

Table S4. Grade 3–4 Exposure-adjusted incidence rates of adverse events in Child-Pugh A and B patients

Grade 3-4 adverse event	Child-Pugh A	Child-Pugh B	p-value
Diarrhoea/colitis	0.03	0.00	0.999
Fatigue	0.19	0.16	0.999
Hepatitis	0.00	0.19	0.138
Arterial hypertension	0.17	0.15	0.999
Hyperthyroidism	0.00	0.19	0.138
Hypophysitis	0.00	0.00	0.999
Hypothyroidism	0.00	0.00	0.999
Mucositis	0.00	0.00	0.999
Palmar-plantar erythrodysaesthesia	0.03	0.00	0.999
Pneumonitis	0.00	0.00	0.999
Proteinuria	0.24	0.00	0.376
Pruritus	0.00	0.00	0.999
Rash	0.00	0.00	0.999

Exposure-adjusted incidence rates (EAIRs), expressed per 100 patient-months, were calculated by dividing the number of patients experiencing at least one adverse event by the total cumulative exposure time from treatment initiation to discontinuation, multiplied by 100. Comparisons of exposure-adjusted incidence rates (EAIRs) between groups were performed using Poisson regression models
